# Association between ambient particulate matter and latent tuberculosis infection among 198 275 students

**DOI:** 10.7189/jogh.14.04244

**Published:** 2024-12-13

**Authors:** Zhongqi Li, Zhan Wang, Peng Lu, Jingxian Ning, Hui Ding, Limei Zhu, Xiaohua Pei, Qiao Liu

**Affiliations:** 1 Department of Chronic Communicable Disease, Center for Disease Control and Prevention of Jiangsu Province, Nanjing, China; 2 Department of Critical Care Medicine, The First Affiliated Hospital of Wannan Medical College (Yijishan Hospital of Wannan Medical College), Wuhu, China; 3 Department of Epidemiology, Center for Global Health, School of Public Health, Nanjing Medical University, Nanjing, China; 4 Division of Geriatric Nephrology, the First Affiliated Hospital of Nanjing Medical University, Nanjing, China

## Abstract

**Background:**

Numerous studies have estimated the impact of outdoor particulate matter (PM) on tuberculosis risk. Nevertheless, whether there is an association between ambient PM and latent tuberculosis infection (LTBI) risk remains uncertain.

**Methods:**

We collected the basic information and LTBI test results of students who underwent freshmen enrolment physical examinations in 68 middle schools from six prefecture-level cities located in eastern China between 2018 and 2021. We also extracted data on air pollutant concentrations and meteorological factors in six cities between 2015 and 2021. We applied the generalised additive model (GAM) to assess the effect of PM on LTBI risk.

**Results:**

We included 198 275 students in the final analysis, of whom 11 721 were diagnosed with LTBI. The LTBI group had higher proportions of males (*P* < 0.001), individuals of Han nationality (*P* < 0.001), and body mass index compared to the non-LTBI group (*P* < 0.001). For each 1-μg/m^3^ increase in PM_10_ concentration, the LTBI risk increased by 0.82% (95% confidence interval (CI) = 0.65–1.00), 0.90% (95% CI = 0.73–1.08), and 0.86% (95% CI = 0.69–1.03) when lagged at one, two, and three years, respectively. For PM_2.5_, the LTBI risk increased by 0.91% (95% CI = 0.63–1.20), 1.05% (95% CI = 0.75–1.36), and 1.32% (95% CI = 0.96–1.69) when lagged at one, two, and three years, respectively.

**Conclusions:**

Outdoor PM concentration was positively correlated with LTBI risk. Considering that many developing countries are facing the dual challenges of high LTBI rates and serious ambient air pollution, reducing outdoor PM concentration would contribute to alleviating their tuberculosis burden.

Tuberculosis remains the leading causes of mortality globally, with nearly 10.6 million new tuberculosis cases and 1.3 million tuberculosis-related deaths reported in 2022 [1]. About one quarter of the world’s population is infected with *Mycobacterium tuberculosis* (MTB), resulting in latent tuberculosis infection (LTBI) [1], meaning that the body has been infected with the bacterium, but has not developed into a state of clinical, i.e. active tuberculosis. Among individuals with LTBI, however, approximately 5–10% will eventually develop active tuberculosis [2], meaning that they present a substantial pool of potential tuberculosis patients. Reducing the risk of LTBI will therefore directly contribute to alleviating tuberculosis burden.

Outdoor particulate matter (PM) pollution has emerged as a significant public health concern due to its association with an increased risk of various diseases. There are two common types of PM pollutants: inhalable PM with an aerodynamic diameter of 10 μm or less (PM_10_) and fine PM with an aerodynamic diameter of 2.5 μm or less (PM_2.5_). Duan et al. [3] demonstrated that for every 10 μg/m^3^ increase in PM_2.5_ concentration, the risk of chronic kidney disease increased by 71%. A meta-analysis indicated that each 10 μg/m^3^ increment in PM_2.5_ concentration resulted in a 66% higher risk of coronavirus disease 2019 (COVID-19) infection [4]. Several studies have also investigated the association between PM and tuberculosis risk. For example, PM_10_ was shown to be positively related to active tuberculosis in Lanzhou [5]. A nationwide study in China reported that both PM_10_ and PM_2.5_ were positively associated with tuberculosis incidence, with relative risks of 1.07 and 1.12, respectively [6]. Another study from Korea revealed that each one standard deviation (5.63 μg/m^3^) increase in PM_10_ concentration was correlated with a 20% increased tuberculosis risk [7]

However, the possible reasons for the above association are unclear. One explanation might be that outdoor PM contributes to tuberculosis risk by increasing LTBI risk; however, research on this link is scarce. We thus performed a time-series study involving students who participated in freshmen enrolment physical examinations in middle schools in eastern China to explore whether ambient PM exposure indirectly contributed to tuberculosis risk by increasing the risk of LTBI.

## METHODS

### Study areas and subjects

Jiangsu Province, located in eastern China, governs 13 prefecture-level cities with a total area of 107 200 km^2^ and a permanent resident population of 85.15 million (end of 2020). Tuberculosis screening in boarding senior and high middle schools in China is required by the National School Tubeculosis Prevention and Control Guidelines [8]. Jiangsu Province began to carry out tuberculosis screening for newly enrolled middle school students in 2017, including tuberculin skin test and chest x-ray examinations.

We included all middle schools in Jiangsu Province that conducted the LTBI test during the 2018–21 freshman enrollment physical examinations as study subjects. This amounted to 68 middle schools from six prefecture-level cities: namely Lianyungang, Taizhou, Wuxi, Xuzhou, Yancheng, and Zhenjiang, all of which are located in different parts of Jiangsu Province (north, central, and south) and have different incidence rates of tuberculosis (**Table 1**).

**Table 1:** Description of the location, economics, tuberculosis burden, and overall population of six cities included in the study in 2023

City	Location	Economics	Incidence of tuberculosis per 100 000 population	Incidence of tuberculosis in students per 100 000 population	Population size in 10 000
Lianyungang	North	Low	23.14	6.35	459.4
Taizhou	Center	Low	24.74	5.97	450.7
Wuxi	South	High	20.07	8.68	749.5
Xuzhou	North	Middle	20.63	7.06	902.0
Yancheng	North	Low	26.00	5.06	668.9
Zhenjiang	South	Middle	24.74	11.19	322.2

### Data collection

We collected the basic information of all students in the form of questionnaires, including sex, age, height (in metres, rounded to two decimal places), weight (in kilograms), ethnicity, tuberculosis history (yes or no), and contact history of tuberculosis patients (yes or no). We subsequently calculated the body mass index (BMI) of each student according to the formula BMI = weight/height^2^.

We also extracted the LTBI test results of all students. The detection of LTBI was done using the purified protein derivative (PPD) method: five IU of PPD is injected intradermally at the center of the anterior one-third of the volar aspect of the left forearm, and the response at the injection site is examined 72 hours later, with skin induration as the criterion. The interpretation of PPD test results typically relies on the average diameter of the induration at the injection site, as well as the presence of accompanying symptoms such as vesicles, necrosis, or ulceration. The criteria for classification are as follows: negative (–), i.e. absence of induration, or an average induration diameter of less than 5 mm; weakly positive (+), i.e. an average induration diameter of 5 mm or greater but less than 15 mm; moderately positive (++), i.e. an average induration diameter of 10 mm or greater, but less than 15 mm; strongly positive (+++ or ++++), i.e. an average induration diameter of 15 mm or greater, or the occurrence of accompanying symptoms such as vesicles, necrosis, and ulceration at the injection site, even if the average induration diameter does not reach 15 mm. According to the national guidelines (WS288-2017), a PPD reaction with an induration diameter of ≥10 mm is defined as LTBI.

**Figure 1:**
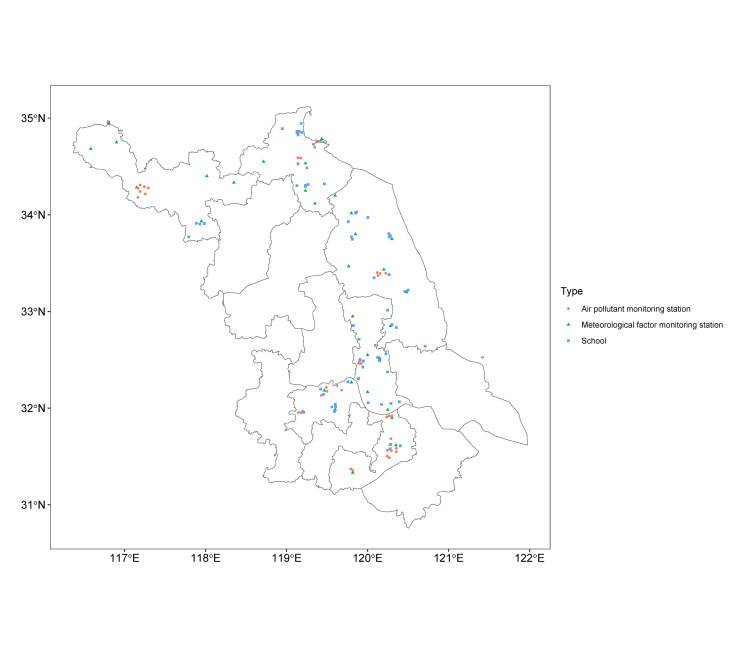
The coordinate diagram of 68 middle schools and all air pollutant monitoring stations and meteorological factor monitoring stations in six cities.

To assess the exposure levels of outdoor PM and meteorological factors for each student, we calculated the distance in kilometres between the 68 schools and the air pollutant monitoring stations and meteorological factor monitoring stations within their city using their longitude and latitude (**Figure 1**). We then used data from the nearest air pollutant monitoring station and meteorological factor monitoring station relative to the school to evaluate the exposure levels of outdoor PM and meteorological factors for students in the school. The analysis included 14 air pollutant monitoring stations and 10 meteorological factor monitoring stations.

Then, we extracted data on outdoor air pollutant concentrations or meteorological factors of the aforementioned monitoring stations in six cities between 2015 and 2021 from the National Urban Air Quality Real-time Release Platform or the China Meteorological Data Sharing Center. These two databases have been used extensively in previous studies [9,10]. Outdoor air pollutant concentrations included PM_10_ (μg/m^3^), PM_2.5_ (μg/m^3^), SO_2_ (μg/m^3^), NO_2_ (μg/m^3^), CO (mg/m^3^), and O_3_ (μg/m^3^), with O_3_ concentration being determined per the daily maximum eight-hour average, while the other air pollutants are assessed based on the daily 24-hour average concentration. Meteorological factors included daily average temperature (°C) and daily average relative humidity (%). If the measurement value of a certain environmental indicator was missing on a certain day, we used the average of the measurement values of the day before and after that date to fill in the missing data.

### Statistical analysis

Previous studies indicated that outdoor PM may have lag effect on tuberculosis risk [11,12]. However, the exact duration of this lag time remains unclear. For example, one study examined the association between outdoor air pollution and tuberculosis risk by setting the maximum lag time at 18 months [13]. Another study investigated the impact of outdoor air pollutants on tuberculosis risk from a lag three months to a lag six months [11]. Notably, only one study to date has explored the link between outdoor PM_2.5_ exposure and LTBI risk at different lag years [14]. Consequently, we calculated different lag times for PM concentration to investigate its potential effect on LTBI risk, namely at one, two years, and three years. The association could therefore be considered robust if it remains significant across different lag times. For instance, if a student participated in the freshmen enrolment physical examination in 2018, the PM exposure level at a lag of one year was the average of PM concentration from 1 September 2017 to 31 August 2018; the PM exposure level at a lag of two years was the average of PM concentration from 1 September 2016 to 31 August 2018, and so on.

The generalised additive model (GAM) extends the common generalised linear model by incorporating smooth terms. After employing the spline function to create smooth terms for the independent variables, not only can their nonlinear effects on the dependent variable be controlled, but also exposure-response curves between independent variables and dependent variable can be plotted. Moreover, by specifying the distribution function of the dependent variable, the GAM can be adapted to various types of data distribution, such as the normal distribution and the binomial distribution. For this reason, the GAM has been extensively utilised in studies on environmental factor exposure and human health [15,16]. Here we used a binomial distribution GAM to evaluate the relationship between PM concentration and LTBI risk. We adjusted the model for the covariates of sex, age, BMI, nation, tuberculosis history, contact history of tuberculosis patients, and average temperature and average relative humidity at the same lag time. We created smooth terms for two meteorological factors using the thin plate spline function with the maximum degree of freedom of two to control their potential nonlinear effects on LTBI risk [17,18]. To quantify the strength of the relationship, we presented the percentage changes in LTBI risk along with their corresponding 95% confidence intervals (CIs) for each one-unit increment in PM concentration. Then, we conducted a subgroup analysis to estimate the association between PM concentration and LTBI risk among males and females, as well as different age groups (divided by median age). We further constructed dual-pollutant models by additionally adjusting each of the four gaseous pollutants in the model in order to assess the stability of the relationship between PM concentration and LTBI risk. Due to the high correlation between outdoor air pollutants, we did not enter them into the model simultaneously to address the multicollinearity problem [15,19].

We conducted all analyses in R, version 4.3.2 (R Core Team, Vienna, Austria). We considered *P*-values <0.05 to be statistically significant.

## RESULTS

### Characteristics of students

After excluding one individual with missing information on height and weight, we included 198 275 students in this study, of whom 11 721 students were diagnosed with LTBI. We found that the LTBI group had a higher proportion of males (*P* < 0.001), individuals of Han nationality (*P* < 0.001), and higher BMI values compared to the non-LTBI group (*P* < 0.001). There were no statistically significant differences in the distribution of tuberculosis history and contact history of tuberculosis patients between the two groups (**Table 2**).

**Table 2:** The characteristics of students (n = 198 275)*

Characteristics	Non-LTBI	LTBI	*P*-value
Sex			<0.001
*Male*	87 229 (46.76)	6115 (52.17)	
*Female*	99 325 (53.24)	5606 (47.83)	
Age, MD (IQR)	15.50 (15.40–15.50)	15.50 (15.40–15.50)	<0.001
BMI, x̄ (SD)	21.43 (2.96)	21.77 (3.08)	<0.001
Nation			<0.001
*Han*	184 650 (98.98)	11 654 (99.43)	
*Others*	1904 (1.02)	67 (0.57)	
TB history			0.347
*No*	186 548 (>99.99)	11 720 (99.99)	
*Yes*	6 (<0.01)	1 (0.01)	
Contact history of TB patients			0.901
*No*	186 527 (99.99)	11 720 (99.99)	
*Yes*	27 (0.01)	1 (0.01)	

BMI – body mass index, IQR – interquartile range, LTBI – latent tuberculosis infection, MD – median, SD – standard deviation, TB – tuberculosis, x̄ – mean

*Presented as n (%) unless specified otherwise.

### Description of air pollutant concentrations and meteorological factors at different lag times

The median concentration of PM_10_ and PM_2.5_ was 67.79 μg/m^3^ (interquartile range (IQR) = 60.23–72.13) and 39.90 μg/m^3^ (IQR = 35.84–46.64) at a lag of one year, and 68.89 μg/m^3^ (IQR = 61.92–76.41) and 42.59 μg/m^3^ (IQR = 38.32–46.60) at a lag of two years, and 71.48 μg/m^3^ (IQR = 65.72–79.21) and 44.37 μg/m^3^ (IQR = 39.97–49.74) at a lag of three years (**Table 3**).

**Table 3:** Description of average air pollutant concentrations and meteorological factors at different lag times

Lag time	Minimum	Maximum	x̄	MD (IQR)
One year				
*PM_10_ (μg/m^3^)*	49.39	120.80	67.99	67.79 (60.23–72.13)
*PM_2.5_ (μg/m^3^)*	29.44	64.19	41.24	39.90 (35.84–46.64)
*SO_2_ (μg/m^3^)*	4.41	16.27	8.61	8.63 (6.05–10.72)
*NO_2_ (μg/m^3^)*	19.34	51.68	28.44	27.69 (23.55–31.94)
*CO (mg/m^3^)*	0.50	1.09	0.73	0.74 (0.65–0.80)
*O_3_ (μg/m^3^)*	93.63	113.49	102.58	102.24 (99.96–104.93)
*Temperature (°C)*	14.60	18.03	16.06	15.78 (15.27–16.93)
*Relative humidity (%)*	69.12	80.81	74.76	74.52 (72.90–76.37)
Two years				
*PM_10_ (μg/m^3^)*	50.52	126.14	70.42	68.89 (61.92–76.41)
*PM_2.5_ (μg/m^3^)*	31.56	65.54	43.05	42.59 (38.32–46.60)
*SO_2_ (μg/m^3^)*	4.61	18.46	9.63	9.03 (7.10–11.79)
*NO_2_ (μg/m^3^)*	20.06	51.68	29.34	28.25 (24.37–33.40)
*CO (mg/m^3^)*	0.52	1.02	0.76	0.76 (0.71–0.84)
*O_3_ (μg/m^3^)*	95.47	112.83	103.61	103.66 (101.05–105.56)
*Temperature (°C)*	14.69	17.88	16.06	15.85 (15.34–16.96)
*Relative humidity (%)*	69.58	80.80	74.97	74.61 (73.17–76.46)
Three years				
*PM_10_ (μg/m^3^)*	54.34	126.61	73.82	71.48 (65.72–79.21)
*PM_2.5_ (μg/m^3^)*	33.87	63.43	44.87	44.37 (39.97–49.74)
*SO_2_ (μg/m^3^)*	5.05	22.39	11.14	11.08 (7.77–13.46)
*NO_2_ (μg/m^3^)*	21.10	51.74	30.34	29.16 (25.66–33.92)
*CO (mg/m^3^)*	0.56	1.20	0.80	0.80 (0.73–0.86)
*O_3_ (μg/m^3^)*	98.25	112.24	104.11	103.58 (102.38–105.38)
*Temperature (°C)*	14.87	17.68	16.02	15.69 (15.23–16.83)
*Relative humidity (%)*	69.81	80.06	75.08	74.86 (73.02–76.69)

IQR – interquartile range, MD – median, x̄ – mean

#### PM_10_ and LTBI risk

**Figure 2:**
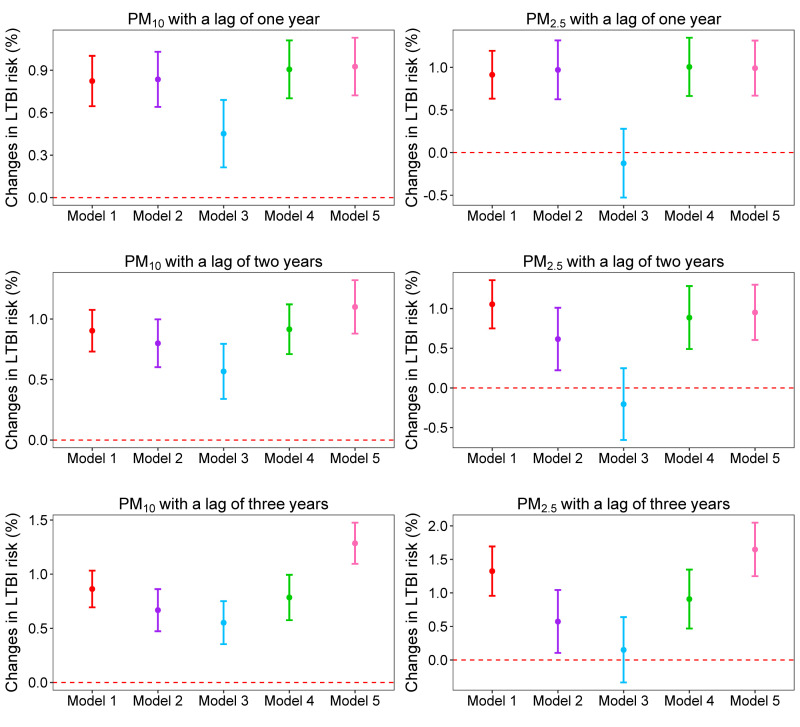
Percentage changes in LTBI risk and their 95% CIs for each one-unit increment in PM concentration. Model 1 was adjusted for sex, age, body mass index, nation, tuberculosis history, contact history of tuberculosis patients, and average temperature and average relative humidity at the same lag time.; Model 2 was based on model 1, additionally adjusted for SO_2_ at the same lag time. Model 3 was based on model 1, but additionally adjusted for NO_2_ at the same lag time. Model 4 was based on model 1, but additionally adjusted for CO at the same lag time. Model 5 was based on model 1, but additionally adjusted for O_3_ at the same lag time. CI – confidence interval, LTBI – latent tuberculosis infection, PM – particulate matter.

For one-unit increment in PM_10_ concentration, the LTBI risk increased by 0.82% (95% CI = 0.65–1.00), 0.90% (95% CI = 0.73–1.08), and 0.86% (95% CI = 0.69–1.03) at lags of one, two, and three years, respectively (**Figure 2**, **Table 4**). The association remained significant when adjusted for SO_2_, NO_2_, CO, or O_3_ at the same lag time in the dual-pollutant models (**Figure 2**). The subgroup analysis showed that PM_10_ concentration positively correlated with LTBI risk among males, females, students <15.50 years old, and students ≥15.50 years old at different lag times (**Table 4**).

**Table 4:** Percentage changes in LTBI risk and their 95% CIs for one-unit increase in PM concentration in subgroups*

	Lag of one year, % (95% CI)		Lag of two years, % (95% CI)		Lag of three years, % (95% CI)	
**Characteristics**	**PM_10_**	**PM_2.5_**	**PM_10_**	**PM_2.5_**	**PM_10_**	**PM_2.5_**
Total*	0.82 (0.65–1.00)	0.91 (0.63–1.20)	0.90 (0.73–1.08)	1.05 (0.75–1.36)	0.86 (0.69–1.03)	1.32 (0.96–1.69)
Sex†						
*Male*	0.78 (0.54–1.02)	0.82 (0.44–1.20)	0.81 (0.58–1.04)	0.99 (0.57–1.41)	0.70 (0.46–0.93)	1.07 (0.57–1.57)
*Female*	0.87 (0.61–1.14)	1.03 (0.62–1.43)	1.00 (0.75–1.25)	1.17 (0.73–1.61)	1.03 (0.80–1.27)	1.59 (1.09–2.09)
Age group in years‡						
*<15.50*	0.62 (0.32–0.92)	0.65 (0.14–1.16)	0.81 (0.50–1.12)	0.82 (0.21–1.43)	0.95 (0.64–1.26)	1.21 (0.49–1.93)
*≥15.50*	0.93 (0.71–1.15)	1.14 (0.82–1.46)	1.02 (0.81–1.22)	1.34 (0.99–1.69)	0.91 (0.70–1.12)	1.68 (1.24–2.12)

CI – confidence interval, LTBI – latent tuberculosis infection, PM – particulate matter

*Models adjusted for sex, age, body mass index, nation, tuberculosis history, contact history of tuberculosis patients, and average temperature and average relative humidity at the same lag time.

†Models adjusted for age, body mass index, nation, tuberculosis history, contact history of tuberculosis patients, and average temperature and average relative humidity at the same lag time.

‡Models adjusted for sex, body mass index, nation, tuberculosis history, contact history of tuberculosis patients, and average temperature and average relative humidity at the same lag time.

#### PM_2.5_ and LTBI risk

A one-unit increment in PM_2.5_ concentration was associated with a 0.91% (95% CI = 0.63–1.20), 1.05% (95% CI = 0.75–1.36), and 1.32% (95% CI = 0.96–1.69) increase in LTBI risk at lags of one, two, and three years, respectively (**Figure 2**, **Table 4**). This relationship remained significant after adjusting for SO_2_, CO, or O_3_ at the same lag time. However, the significance disappeared when adjusted for NO_2_ in the dual-pollutant model (**Figure 2**). In the subgroup analysis, PM_2.5_ concentration was positively related to LTBI risk among males, females, students <15.50 years old, and students ≥15.50 years old at different lag times (**Table 4**).

## DISCUSSION

To our knowledge, this is the largest-scale and one of the first studies investigating the association between PM and LTBI risk. We observed a positive link between ambient PM concentration and LTBI risk.

Although several studies have explored the impact of outdoor PM on tuberculosis risk [6,11,12], the cause for this association remains unknown. One plausible explanation is that outdoor PM exposure may contribute to LTBI, which in turn leads to active tuberculosis. To date, only one study, conducted among 4790 older adults residing in 17 villages in rural China between 2013 and 2018, investigated the impact of ambient PM exposure on LTBI risk [14]. Its authors used a logistic regression model to estimate the association between PM_2.5_ exposure and LTBI risk and found no significant relationship. Several factors may account for this result. First, compared with previous studies on PM exposure and health, they neither included relative humidity as a covariate in their final analysis, nor controlled the potential nonlinear effect of weather conditions on LTBI [15,20,21]. Second, older adults, who formed the population of the aforementioned study, tend to have long-term exposure to outdoor air pollution and therefore may have lower sensitivity to changes in PM concentration compared to younger populations. This reduced sensitivity could have contributed to the lack of significant association between PM_2.5_ exposure and LTBI risk in this particular population. Furthermore, out of the 4790 subjects in this study, 1506 were diagnosed with LTBI according to the interferon-γ release assay (QuantiFERON, QFT). The observed LTBI rate of 31.44% in the aforementioned study is notably higher than previously reported rates ranging from 13% to 20% based on QFT in rural China [22]. This high LTBI rate among the study population may have led to an underestimation of the influence of PM_2.5_ exposure on LTBI risk, as the high prevalence of LTBI could overshadow any incremental effect of PM_2.5_ exposure.

Here we assessed the association between outdoor PM concentration and LTBI risk among a large sample of students and found that both PM_10_ and PM_2.5_ were positively correlated with LTBI risk at different lag times. We also found that the positive link between PM_10_ and LTBI risk was robust after adjusting for each of the other four gaseous pollutants, but the relationship between PM_2.5_ and LTBI risk was no longer significant when adjusted for NO_2_. This may suggest the possibility of a synergistic effect between PM_2.5_ and NO_2_, wherein the presence of NO_2_ can enhance the effect of PM_2.5_ on LTBI. As NO_2_ has certain irritancy and has the capacity to react with water, yielding nitric acid, which can subsequently damage the respiratory mucous membrane. This process facilitates the penetration of both PM_2.5_ and MTB into the lungs, thereby enhancing the risk of infection [23]. Therefore, after controlling the impact of NO_2_, we found no significant association between PM_2.5_ and LTBI risk. The influence of PM_2.5_ exposure exclusively on LTBI need to be explored in further studies. Additionally, the positive link was significant between different student populations at different lag times. Moreover, we observed that the influence of PM_2.5_ on LTBI risk was slightly stronger than PM_10_. This discrepancy may be attributed to the smaller particle size and longer suspension time of PM_2.5_ compared to PM_10_. These characteristics allow PM_2.5_ to not only penetrate deep into the lungs but also enter the circulatory system by crossing the respiratory barrier [24]. As a result, PM_2.5_ poses a greater threat to health.

The mechanism for the role of outdoor PM in LTBI risk was beyond the scope of our study. However, several potential biological explanations may be primarily related to immune dysfunction. First, the respiratory tract can produce secretions to wrap MTB and then clear it to defend against MTB invasion. Exposure to PM may weaken the mucociliary clearance of airway secretions, thus raising the possibility of MTB infection [25]. Second, alveolar macrophages (AMs) are the first line of immune defense in the lungs, which can eliminate harmful bacteria. Nevertheless, PM, particularly PM_2.5_, can reach the alveolar region and weaken the activity of AMs. Specifically, PM exposure is likely to reduce the expression of phagocytosis-related receptors on the surface of AMs, impairing the ability of AMs to phagocytose MTB [26]. Third, exposure to PM decreased the expression of cytokines (tumor necrosis factor-α (TNF-α), interferon-γ (IFN-γ), etc.) in peripheral blood mononuclear cells (PBMCs), suppressing the role of PBMCs in phagocytosing MTB and controlling MTB growth [27,28]. Moreover, exposure to diesel exhaust containing PM has been reported to reduce the expression of several cytokines, such as TNF-α and IFN-γ in murine lung tissues [29]. Specifically, TNF-α exhibits a key role in suppressing MTB infection by augmenting the capacity of macrophages to phagocytise and eliminate MTB. Its production is also crucial for the formation of granulomas, which act as a means to sequester MTB and thereby prevent its dissemination [30]. Meanwhile, IFN-γ is capable of inducing macrophage activation, inducing them to attack and eradicate MTB [31]. Larger studies investigating LTBI risk, PM exposure, and immunological measures are warranted to provide further insights into the underlying mechanisms behind the observed strong association in our research.

This study has several potential limitations. First, we calculated and compared the straight-line distance between each school and several environmental monitoring stations around the school to determine the closest such station, after which we used its data to evaluate the outdoor PM exposure level of each student. This approach may not accurately reflect the actual levels of students’ outdoor PM exposure, possibly introducing exposure misclassification and thereby leading to an underestimation of the observed risk. Future investigations into the impact of PM exposure on LTBI necessitate the employment of more precise methodologies for assessing individual exposure level to air pollutants. Second, the accuracy of the PPD method in detecting LTBI was affected by Bacillus Calmette-Guerin vaccination and the *Nontuberculous Mycobacterium* infection, leading to false-positive test results. This means that the PPD method may overestimate the prevalence of LTBI among the student population, thereby distorting the true association between PM exposure and LTBI risk. More precise diagnostic tools for LTBI, such as the QuantiFERON-TB assay, could help with more accurately exploring the impact of PM exposure on LTBI. Third, we did not consider other confounders which impact LTBI risk (e.g. secondary comorbidities (diabetes, etc.), socioeconomic status) as they were not available in our database. Factors such as diabetes, unhealthy lifestyles and hygienic practices, and exposure to indoor air pollution may compromise the immunity level of individuals, thereby working together with outdoor PM exposure to elevate the risk of contracting MTB infection. It is therefore crucial that these factors be thoroughly considered in future studies analysing the impact of outdoor PM on LTBI risk. Fourth, we only estimated the effect of outdoor PM exposure on LTBI risk among middle school students, so the positive association we observed here need to be validated in the wider population.

## CONCLUSIONS

Our results indicated a positive association between outdoor PM concentration and the risk of LTBI. Given that numerous developing countries are facing the dual challenge of high LTBI rates and severe ambient air pollution, reducing outdoor PM concentration could help alleviate their tuberculosis burden.
